# RNA-Seq transcriptome profiling of immature grain wheat is a technique for understanding comparative modeling of baking quality

**DOI:** 10.1038/s41598-024-61528-y

**Published:** 2024-05-13

**Authors:** Hossein Ahmadi-Ochtapeh, Hassan Soltanloo, Seyyede Sanaz Ramezanpour, Ahad Yamchi, Vahid Shariati

**Affiliations:** 1 Crop and Horticultural Science Research Department, Agricultural Research, Education and Extension Organization (AREEO), Golestan Agricultural and Natural Resources Research and Education Center, Gorgan, Iran; 2 Plant Breeding and Biotechnology Department, Gorgan University of Agricultural Sciences and Natural Resources (GUASNR), Gorgan, Iran; 3 Department of Plant Molecular Biotechnology, Assistant Professor in National Institute of Genetic Engineering and Biotechnology, Karaj, Iran

**Keywords:** RNA-Seq, qRT-PCR, Transcriptome, Gene ontology, Glutenin subunit, Biotechnology, Plant biotechnology

## Abstract

Improving the baking quality is a primary challenge in the wheat flour production value chain, as baking quality represents a crucial factor in determining its overall value. In the present study, we conducted a comparative RNA-Seq analysis on the high baking quality mutant “O-64.1.10” genotype and its low baking quality wild type "Omid" cultivar to recognize potential genes associated with bread quality. The cDNA libraries were constructed from immature grains that were 15 days post-anthesis, with an average of 16.24 and 18.97 million paired-end short-read sequences in the mutant and wild-type, respectively. A total number of 733 transcripts with differential expression were identified, 585 genes up-regulated and 188 genes down-regulated in the “O-64.1.10” genotype compared to the “Omid”. In addition, the families of HSF, bZIP, C2C2-Dof, B3-ARF, BES1, C3H, GRF, HB-HD-ZIP, PLATZ, MADS-MIKC, GARP-G2-like, NAC, OFP and TUB were appeared as the key transcription factors with specific expression in the “O-64.1.10” genotype. At the same time, pathways related to baking quality were identified through Kyoto Encyclopedia of Genes and Genomes. Collectively, we found that the endoplasmic network, metabolic pathways, secondary metabolite biosynthesis, hormone signaling pathway, B group vitamins, protein pathways, pathways associated with carbohydrate and fat metabolism, as well as the biosynthesis and metabolism of various amino acids, have a great deal of potential to play a significant role in the baking quality. Ultimately, the RNA-seq results were confirmed using quantitative Reverse Transcription PCR for some hub genes such as alpha-gliadin, low molecular weight glutenin subunit and terpene synthase (gibberellin) and as a resource for future study, 127 EST-SSR primers were generated using RNA-seq data.

## Introduction

Wheat (*Triticum aestivum* L., 2n = 6x = 42) is an important food crop in the world and possesses unique flour quality that can be used to make various food products^1^. However, current wheat varieties need improvement in processing quality to meet the increasing demand for better quality food products^2^. Processing quality of wheat flour is determined by grain protein concentration and its composition which confer wheat dough with unique rheological properties, making it possible to produce a series of quality foods for human consumption^3–5^. Baking quality is one of the most important parameters throughout the value chain of wheat flour^6^. Seed is the primary storage organ in plants for storing nutrients such as starch, lipids, and proteins^7^ and the baking quality is largely influenced by these nutrients. The content of gliadins and glutenins^6^, optimal water absorption, rheological parameters^8^ and grain hardness^9^ are correlated to high baking quality. Determination of flour water absorption, as well as the results of the farinographic analysis of the flour play a key role in the assessment of wheat flour baking quality ^10^. However, improving baking quality is a challenge for wheat breeders due to the time-consuming and costly testing forcing breeders to postpone sophisticated quality tests to the very last phases of variety development^11^. The genome of a plant is the most critical factor to control baking quality trait in wheat^12^. For example,^2^ processing quality related key genes such as glutenin and gliadins, puroindolines, grain softness protein, alpha and beta amylases, proteases, were identified, and many other candidate genes related to cellular and molecular functions. Therefore, an understanding of the molecular basis of this trait would be a major advantage in wheat breeding^13^. Advances in molecular techniques offer new opportunities to identify the genetic and molecular basis of bread quality in wheat^1,2,14^.

RNA-Seq analysis of various wheat genotypes has opened up new avenues for research on grain development and its composition, which ultimately determines the nutritional and functional quality of wheat^15,16^. The gene expression during wheat grain development plays a crucial role in determining the yield and nutritional properties of the crop. Identifying genes expressed during the grain filling stage can be useful for improving yield and grain quality^17,18^. Several studies have applied transcriptomics approaches to investigate the gene expression during grain development stages in wheat^2,15–17,19–21^. The grain development process of wheat can be categorized into three stages, including cell division and expansion (0 ~ 14 DPA (days post-anthesis)), effective grain filling (14 ~ 28 DPA), and maturation and desiccation (28 DPA to maturity)^22^. Understanding these stages and the associated gene expression can lead to the development of new and improved wheat varieties with superior nutritional and functional properties^17,18^. Early grain development, specifically the first 14 days after pollination, is critical for the final yield and quality of wheat. During this time, rapid grain expansion occurs through continuous cell division and endosperm filling with starch and gluten^16,23^. At 30 DPA, a diverse range of genes are expressed at low levels with a predominance of genes associated with seed defense and stress tolerance^13^.

Wheat flour quality is highly influenced by protein content and composition, with glutens being the most abundant storage proteins, comprising about 80% of total grain proteins. Glutenins consist of high and low-molecular-weight glutenin subunits (HMW-GS and LMW-GS). Gene expression from these protein families occurs early in grain development^16,23^. Zamani et al.^24^ and Izadi-Darbandi et al.^25^ reported that mutant, “O-64.1.10” and its parent, “Omid” wheat cultivar have two HMW-GS genes, Dx2 + Dy12 and Bx7 + By8 but the improvement of baking quality in the mutant, “O-64.1.10” was unknown. A negative effect of Dx2 + Dy12 was published previously by several studies^26–31^. The allelic pair Bx7 + By8* (Glu-B1a1) strongly associated with dough strength^32^. The complex processing quality of wheat is controlled by many genes, which have not been completely explored^2^. For example, phytohormones are the predominant biochemical basis for grain morphogenesis^33^ and reported to be important signals in controlling seed development, maturation, and nutrients accumulation^34^. Gene ontology (GO) enrichment and pathway enrichment analysis showed that many transcription products and transcription factors (TFs) associated with carbohydrate and protein metabolism were abundantly expressed in the grain^35^. TFs regulate target genes to ensure tightly regulated developmental process^15^. bZIP TFs are regulators of important plant processes such as seed storage protein gene regulation^36^, energy metabolism^37^, unfolded protein response^36^, and hormone and sugar signalling^38^. NAC TF play a significant role in regulating the accumulation of glutenin and starch in wheat endosperm, which are crucial for the grains quality^39^.

Rahemi et al.^40^ reported that water absorption percentage, valorimeter value, farinograph quality number, zeleny number, hardness, wet gluten, protein content in high baking quality mutant “O-64.1.10” exhibited greater than “Omid”. We compared RNA-Seq data from immature grains of a “O-64.1.10” wheat genotype and its wild type cultivar, "Omid," which have significantly different baking qualities. The purpose of our study was to identify genes that are primarily expressed in wheat grain and are related to baking quality. Through this study, we gained new insights into the molecular mechanisms that underlie seed quality and identified potential candidate genes related to wheat grain quality. Our findings provide a solid foundation for future research aimed at improving bread-making quality.

## Material and methods

At all stages, the research complied with relevant institutional, national, and international guidelines and legislation.

### Plant materials and sampling

To investigate the transcriptome of immature seeds of *Triticum aestivum* L, mutant “O-64.1.10” genotype and its low baking quality wild type "Omid" cultivar were obtained from the GenBank of Nuclear Science and Technology Research Institute in Karaj, Iran. We used two genotypes of Iranian bread wheat, namely “O-64.1.10” (with high baking quality) and its parent cultivar “Omid” (with poor baking quality), to conduct a comparative analysis of their baking quality (Table 1). Mutant genotype “O-64.1.10” was produced by the gamma irradiation approach and evaluated for baking quality through rheological and proteomics trials^25,40^. The “Omid” and its mutant “O-64–1-10”, were cultivated at the research field of Gorgan University of Agricultural and Natural Resources Gorgan, Iran. The main stem heads were marked with the anthesis date. The grains from the middle ear were collected at 5, 10, 15, 20, and 30 DPA between 9:00 and 10:00 am. For dynamic comparison of developing grains three biological replications of samples were used for the five stages. Also for RNA-seq samples, the seeds of “Omid” and “O-64.1.10” were used at 15 DPA. For any genotypes the seeds of 9 spikes (10 grains per spike) harvested from 9 different plants (three plants per three replications) and pooled. The samples were promptly frozen in liquid nitrogen and stored at − 80 °C for subsequent total RNA extraction.

**Table 1:** Comparison the baking quality traits in mutant genotype “O-64.1.10” and its wild type “Omid” cultivar^12^.

Genotype	Protein content	Wet gluten	Hardness	Zeleny number	Valorimeter value	Water absorption percentage	Farinograph quality number	Bread volume
Omid	11.5^a^	20.5^b^	51^b^	31^b^	41^b^	57.65^b^	41.25^a^	568. 5^a^
O-64.1.10	13^a^	36^a^	54.5^a^	36.5^a^	54^a^	66.75^a^	75^a^	505. 5^b^

^a, b^Mean values marked with different letters are significantly different (*p* ≤ 0.05) by LSD test.

### RNA isolation and cDNA library construction

We utilized 100 mg seed samples at 15 DPA and p-Biozol Buffer (BioFlux, Japan) to extract total RNA, following the manufacturer's recommendations. Additionally, 1% (w/v) RNase-free agarose gel electrophoresis and a Nanophotometer (Implen, Germany) were used to evaluate the amount and quality of RNA samples. The RNA samples of 15 DPA, passing the quality and quantity control, were sent to Beijing Genomics Institute (BGI), Hong Kong, China for cDNA library construction and sequencing on the Illumina sequencing platform (Illumina HiSeq™ 2500). A comprehensive quantitative evaluation of each RNA samples was performed using a Nanodrop 8000 Spectrophotometer (ThermoScientific, USA) and an Agilent 2100 Bioanalyzer System (Agilent Technologies, USA) to produce information on RNA concentration. The high-quality RNA (OD 260/280 = 2.06–2.08; OD 260/230 = 1.9–2.09; RIN value 7.5) was further processed for cDNA library creation using the Illumina TrueSeq RNA Sample Prep kit. To produce 150 bp paired-end reads, NGS sequencing of cDNA from seed tissue was performed.

### Data processing and analysis

The quality of paired-end reads from raw sequencing data was assessed both before and after trimming. The sequencing quality of the raw reads from each sequenced sample was assessed using FASTQC software (https://www.bioinformatics.babraham.ac.uk/projects/fastqc/) accessed on January 8th, 2019)) and CLC Genomic Workbench 7.5.1 (CLC Bio-Qiagen, Denmark). Trimmomatic software (version 0.36) was used to remove adapter sequences, low-quality nucleotides/sequences, and reads shorter than 150 bp. The remaining high-quality reads with a Phred score of ≥ 30 was used for downstream analyses. These reads were aligned against the wheat reference genome (version IWGSC RefSeq v1.0, http://plants.ensembl.org/Triticum_aestivum/) using Hisat2 software (version 2.2.1.0)^41^. The mapped reads from each sample were assembled using Cufflinks v2.0.2 and htseq. These Cufflink assemblies were merged using Cuffmerge. The outputs were then used for differential expression analysis by Cuffdiff and EdgeR packages^42^. The normalization of gene expression values of two samples was estimated as fragments per kilobase of transcript per million fragments mapped (FKPM) and fold change (FC). Data analysis with an adjusted p-value threshold of 0.001 and Log2 Fold Change (log2FC) of ≥ 2 were assigned as differentially expressed genes (DEGs). GO enrichment analysis for DEGs was performed using goseq and AgriGO version 2.0. The Benjamini and Hochberg approach for controlling the false discovery rate (FDR) was used to adjust P-values. The threshold of 0.01 was set for the FDR value. The GO analysis was carried out to functionally categorize the DEGs from 15 DPA into three major aspects: "cellular component (CC)," "molecular function (MF)," and "biological process (BP)". Pathway enrichment analysis of DEGs was performed using the KEGG (Kyoto Encyclopedia of genes and genomes) database (http://www.genome.ad.jp/kegg/). To identify TFs, transcriptional regulators (TRs), and protein kinases (PKs) encoding genes, The DEGs were screened using the Plant Transcription Factor & Protein Kinase Identifier and Classifier database (iTAK v1.6). Simple Sequence Repeats (SSRs) were identified from RNA-seq data using the Perl script of MISA (MIcroSAtellite identification tool)^43^ with default parameters^44^.

### Validation of RNA-seq data by real-time PCR

The RNA-Seq results were validated through the use of quantitative Reverse Transcription PCR (qRT-PCR). The qRT-PCR was performed on three genes that were chosen from the DEG analysis. The primers were designed based on the 3́-UTR region of the sequence (Table 2) using the Primer3 online software (http://www.embnet.sk/cgi-bin/primer3_www.cgi)^45^.

**Table 2:** List of primers used in the experiment for quantitative Reverse Transcription PCR amplification.

Primer name	Gene ID	Primer sequence		Tm	GC%	Product size (bp)
LMW-GS	TraesCS1B01G011600.1	Forward	5ʹ- ACAACAGGTTCAGGGTTCCA-3ʹ	59.08	50	158
		Reverse	5ʹ- CTATCTGGTGTGGCTGCAAA-3ʹ	58.17	50	
Alpha-gliadin	TraesCSU01G160200.1	Forward	5ʹ- ATGTTGTCAGCAGTTGTGGC-3ʹ	59.33	50	154
		Reverse	5ʹ- TTACTGAGGCTGCTGGTAGG-3ʹ	58.80	55	
Gibberellin	TraesCSU01G099900.1	Forward	5ʹ- TGGACGAGAGAATTGAGGCA-3ʹ	58.73	50	152
		Reverse	5ʹ- ATCGAACACATGGGGAGGG-3ʹ	59.08	57.9	
GAPDH	AK359500.1	Forward	5'-GTTGGCAAGGTGCTCCCAGA-3'	62.70	60	121
		Reverse	5'-GCTCATAGGTGGCTGGCTTG-3'	61.10	60	

Before first-strand cDNA synthesis, possible genomic DNA contamination was removed by RNase-free DNaseI (Thermo Scientific, USA) at a ratio of 1 U DNaseI to 2 µg RNA, 1X DNaseI buffer, 10 U Ribolock RNase inhibitor (Thermo Scientific, USA), and DEPC water up to 9 µl, followed by incubation at 37 °C for 30 min. To finalize DNaseI activity, 25 mM EDTA buffer was added to the reaction and the RNA was then heat-denatured at 65 °C for 10 min. First-strand cDNA synthesis was carried out using 1 µg of total RNA in the reaction mixture containing 0.5 µg Oligo (dT) primer and DEPC water (nuclease-free) up to 11 µl. To eliminate secondary structures, the liquid was gently mixed, quickly centrifuged, then incubated at 70 °C for 5 min. Then, 4 µl 5X cDNA reaction buffer, 10 mM dNTP mix, and 20 U Ribolock RNase inhibitor were added to the reaction mixture. The final volume was adjusted to 19 µl with DEPC-treated water, and the mixture was incubated for 5 min at 37 °C. 200 U Revert Aid enzyme (Thermo Scientific, USA) was added to the reaction mixture and incubated at 42 °C for 1 h. A qRT-PCR was performed using an iCycler thermal cycler (Bio-Rad, iQ5, USA) with a reaction volume containing 3 µl of diluted cDNA, 10 µl of 2X SYBR Bio Pars PCR Master Mix (Gorgan University of Agricultural Sciences and Natural Resources, Iran), and 1 µl of each gene-specific primer (10 pmol) in a final volume 20 µl with double distilled water. The time course of qRT-PCR were done at the following conditions: 3 min at 95 °C for 1 cycle; 10 s at 95 °C, 10 s at 62 °C and 10 s at 72 °C for 35 cycles, and 2 min at 72 °C for 1 cycle. Upon that, PCR was done on each sample in three technical and biological replications. The fluorescence signal was detected at 72 °C. The REST software was used for the gene expression analysis^46^, and the relative expression was computed utilizing the comparative Ct (2-∆∆^Ct^) method^47^ and compared with the expression levels of RNA-Seq. For qRT-PCR normalization, glyceraldehyde 3-phosphate dehydrogenase (GAPDH) was utilized as the reference gene.

### Gene ontology analysis

The major biological activities of DEGs were determined using a GO enrichment analysis. DEGs from the 15 DPA stage were annotated and grouped into three major groups based on their functional characteristics: CC, MF, and BP categories.

## Result

### Grain morphology changes during seed development

**Figure 1:**
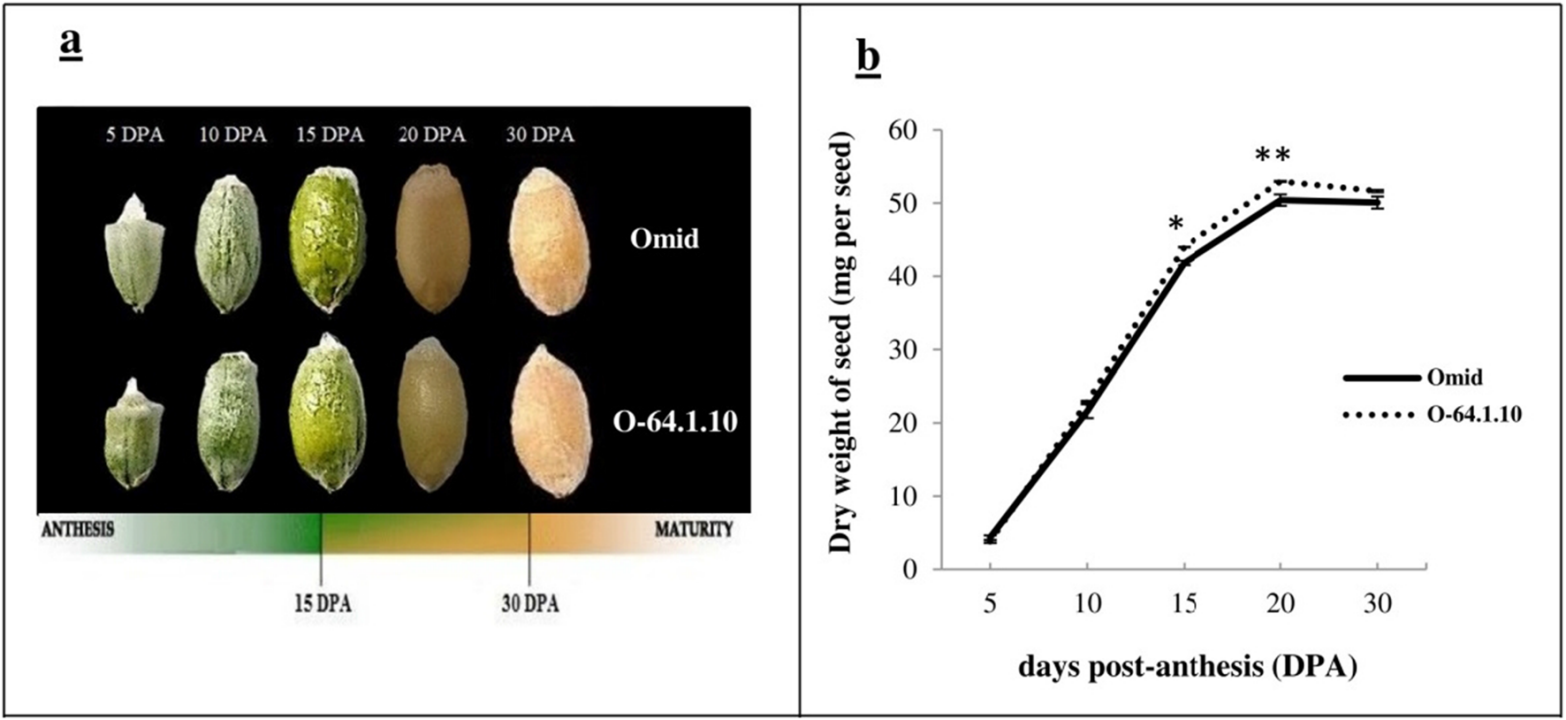
Dynamic comparison of grains at different developmental stages between in high baking quality mutant “O-64.1.10” and its wild type genotypes “Omid”. (**a**) Changes in grain morphology at 5, 10, 15, 20 and 30 DPA of “O-64.1.10” and “Omid”. (**b**) Changes in dry grain weight during the development process. Three biological replications and t-test were done to obtain the curve. *—significant at *P* < 0.05 and **—significant *P* < 0.01. I: standard error.

The dynamic comparison of developing grains was examined at 5, 10, 15, 20, and 30 DPA stages between the “O-64.1.10” genotype and its “Omid” counterpart (Fig. 1a). The dry weight of the seeds showed a continuous increase from 5 to 20 DPA and reached its highest point at 20 DPA in both the mutant genotype “O-64.1.10” and its parent genotype “Omid” (Fig. 1b). Our results showed that mutant “O-64.1.10” exhibited greater grain weight growth than “Omid” at15, 20 and 30 DPA. Also highest rate of dry weight accumulation per day occurred between 10 and 15 DPA. The 15 DPA stage are beginning of effective grain filling stage in this stage the grain dry weight increases by about two-fold. The 15 DPA stage for the RNA-seq analysis. This finding is in agreement with earlier study conducted by Shewry et al.^12^.

### Identification of differentially expressed genes

**Figure 2:**
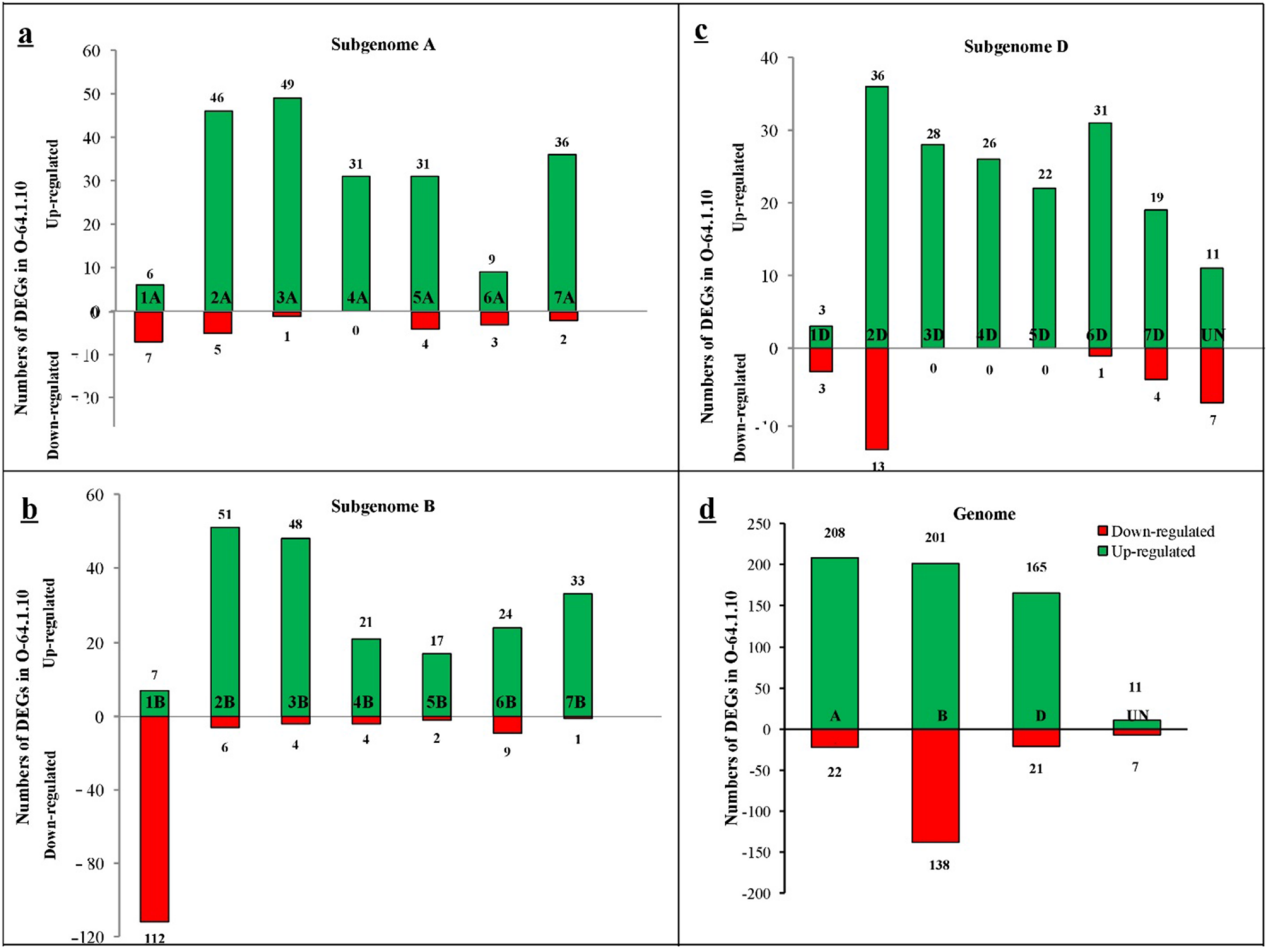
The numbers of DEGs on the A, B and D genomes in the “O-64.1.10” genotypes relative to the “Omid” cultivar. UN: unassigned chromosome.

The quality of RNA and quality of high-throughput sequencing data showed in supplementary data (S1 Figure). A total of 18.97 and 16.24 million paired-end short-read sequences with high-quality (Q30 > 99%) obtained by Illumina sequencing of the “Omid” and “O-64.1.10” cDNA libraries, respectively. The GC (guanine-cytosine) content for the clean data was 51% and 52% for the “Omid” and “O-64.1.10”, respectively (Table 3). DEGs were found in 773 genes between “O-64.1.10” and “Omid” (supplemental data 1). 585 genes up-regulated and 188 genes down-regulated in the “O-64.1.10” genotype compared to the “Omid”. Our study showed that most of the DEGs (339 genes, 43.85%) were anchored on B-genome (Fig. 2d, supplemental data 2). In addition, we found that up-regulated genes in “O-64.1.10” were enriched on chromosomes 2A (5.95%) and 3A (6.33%) and chromosomes 2B (6.59%) and 3B (6.2%), whereas down-regulated genes in “O-64.1.10” were overrepresented on chromosome 1B (14.5%) (Fig. 2).

**Table 3:** Summary of high-throughput sequencing data in “Omid” and mutant at 15 DPA.

Sample	Sequence length (base pairs)	Number of sequences (total reads)	Total nucleotides in data set (base)	% sequence duplication levels	% GC-content	^a^Q30 (%)
Omid	150	18,976,039	2,846,405,850	0.5335	51	99.78
O-64-1-10	150	16,246,625	2,436,993,750	0.672	52	99.73

^a^paired-end short-read sequences with high-quality (Q30≻ 99%).

### Functional classification and gene ontology analysis

**Figure 3:**
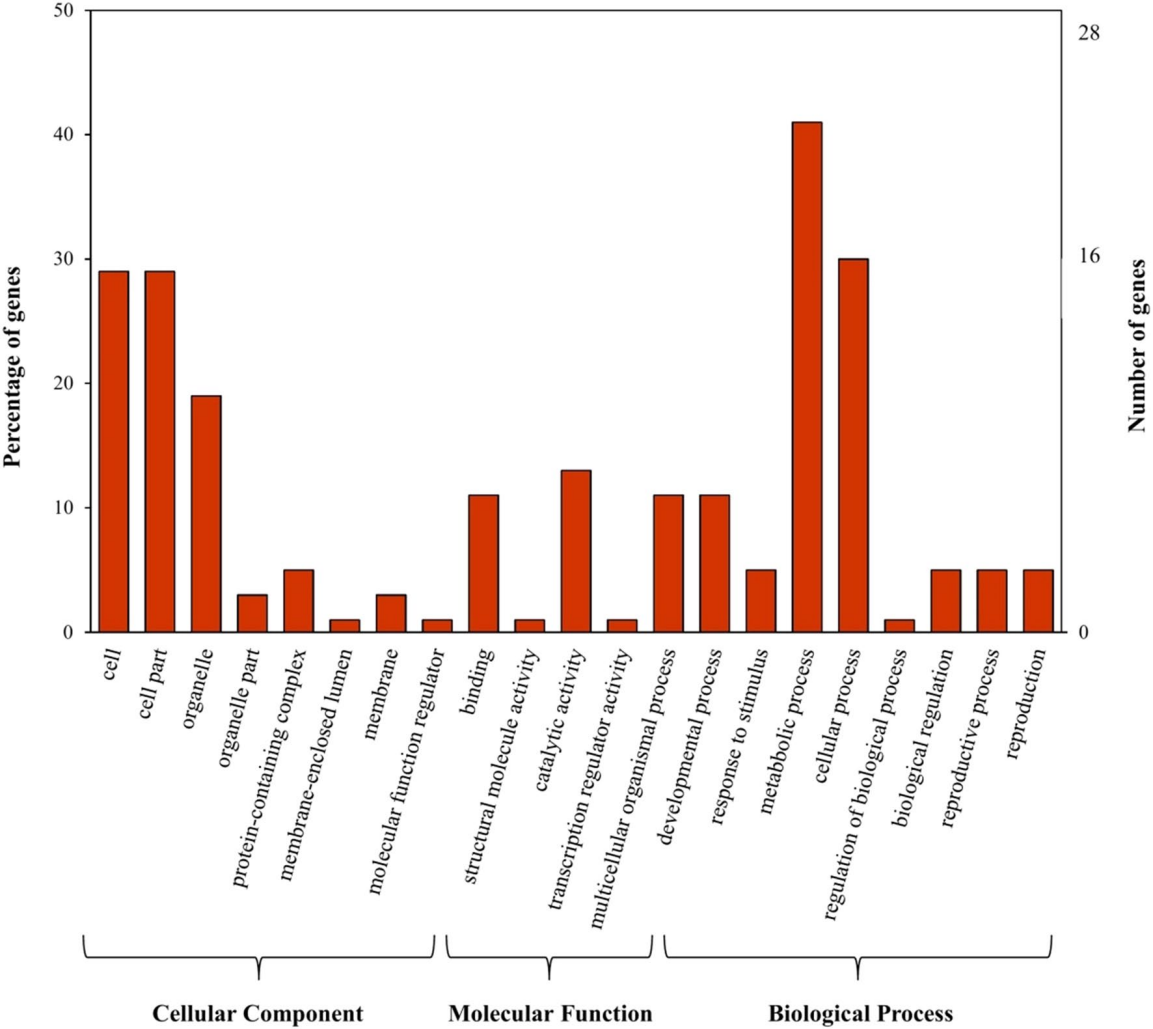
Histogram of Gene Ontology (GO) classifications of DEGs between “Omid” and “O-64.1.10” genotypes in 15 DAP. The results are summarized in three principal GO functional categories: biological processes, cellular component, and molecular function.

GO enrichment analysis was carried out to characterize the main biological functions of DEGs in seeds 15 DPA. The DEGs from 15 DPA stage were functionally annotated and classified ontologically into three principal categories: CC, MF, and BP. Furthermore, 62 GO terms were enriched in the DEG from O-64.1.10 and Omid, including 19 CC, 15 MF, and 28 BP. A total of 733 DEGs were assigned to three main GO functional categories and then divided into 21 sub-categories. Metabolic process, cellular process, developmental process, and multicellular organismal process were found to be dominant in the BP category. The MF category was primarily dominated by catalytic activity and binding. Cell, cell part, and organelle were foremost in the cellular CC category (Fig. 3). The “O-64.1.10” genotype had the highest number of genes involved in specific BP, such as cellular protein catabolic process, diterpenoid biosynthetic process, gibberellin metabolic process, cellular nitrogen compound biosynthetic process, cellular amide metabolic process, lipid biosynthetic process and response to abiotic stimulus. Overall, these results suggest that the genes associated with wheat early grain development play crucial roles in encoding diverse regulators and proteins.

**Figure 4:**
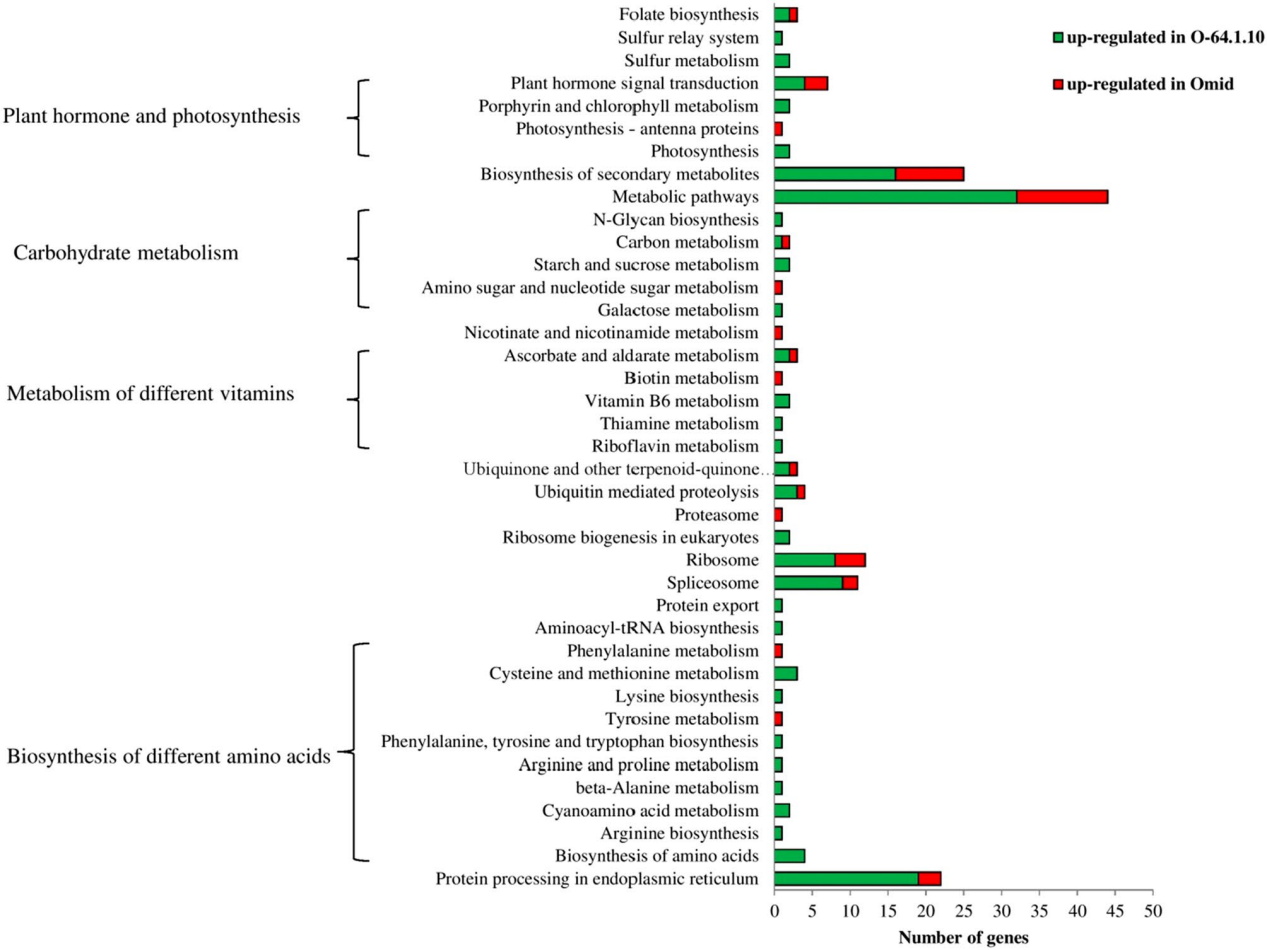
Bar diagrams displayed the KEGG significantly metabolic pathways in the “O-64.1.10” and “Omid” at 15 DPA.

At the same time pathway enrichment analysis using KEGG database was performed to reveal the active biological pathways involved in baking quality of immature wheat grains. In this study, 68 and 34 pathways were identified in the “O-64.1.10” and the wild-type, respectively. Among all the pathways, the largest pathways were metabolic pathway, protein processing in endoplasmic reticulum and biosynthesis of secondary. An analysis using the KEGG database on biological pathways in the “O-64.1.10” genotype showed that a total of 187 annotated DEGs were assigned to 68 pathways. Among the 68 pathways, the largest pathway was metabolic pathways, which contained 32 genes, followed by protein processing in endoplasmic reticulum (19 genes), biosynthesis of secondary metabolites (16 genes), spliceosome (9 genes), ribosome (8 genes), plant hormone signal transduction (4 genes), and biosynthesis of amino acids (4 genes). The KEGG analysis in the “O-64.1.10” genotype indicated that 39 DEGs were involved in protein processing in endoplasmic reticulum (19 genes), biosynthesis of different amino acids pathways (14 genes), and different vitamins metabolism pathways (6 genes), which might participate in regulating baking quality (Fig. 6). Furthermore, the carbohydrate metabolism pathways (5 genes) in the wild-type genotype might participate in regulating baking quality. Pathway enrichment analysis of “Omid” genotype displayed the total of 68 annotated DEGs were assigned to 34 pathways. The top three enriched pathways include metabolic pathways (12 genes), biosynthesis of secondary metabolites (9 genes), and ribosome (4 genes) were largest pathways (Fig. 4, Supplemental data 4).

**Figure 5:**
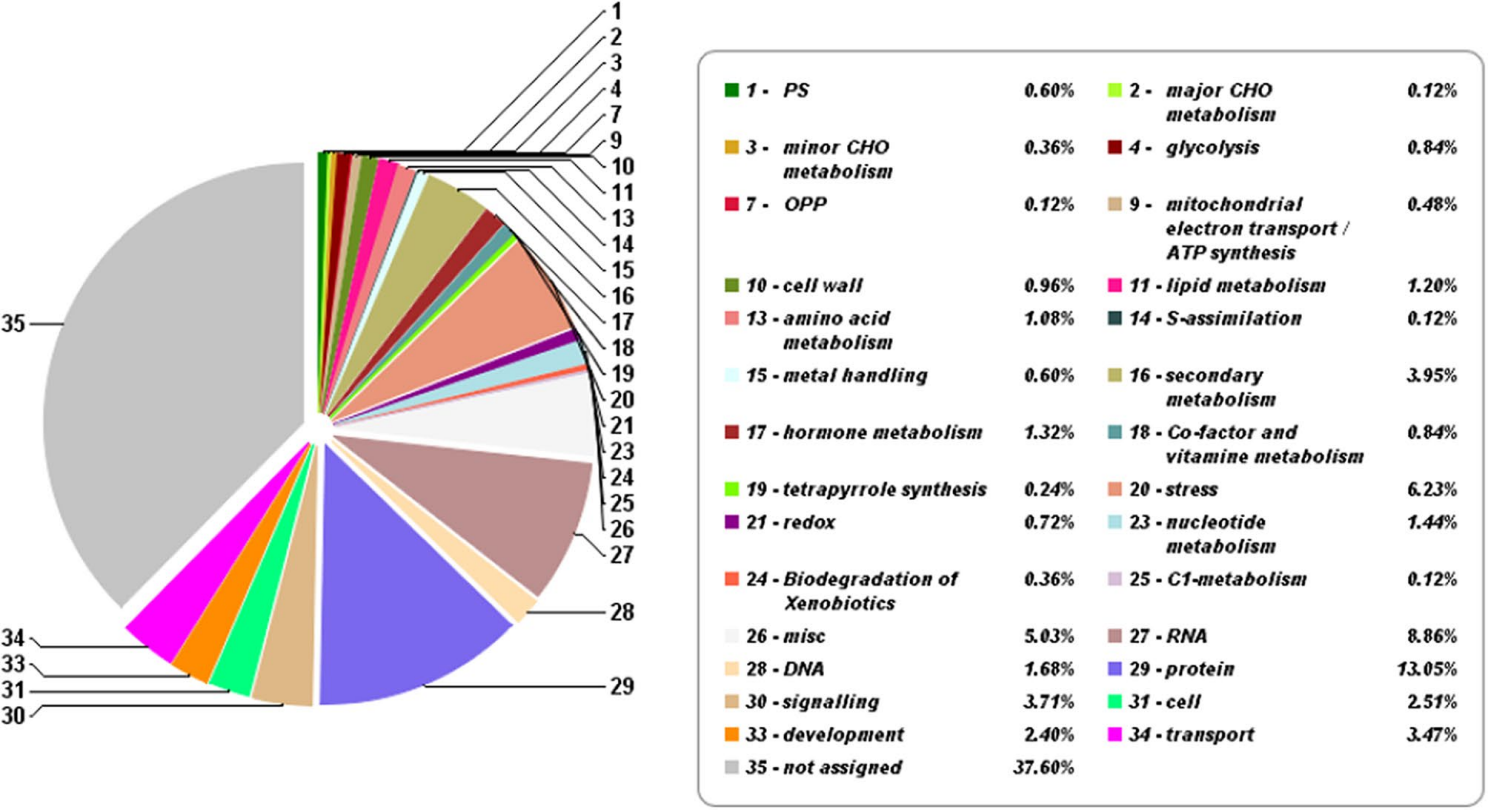
The amount of hub genes expression in 35 segregate biological groups (35 main BINs) using the Mercator web server.

Functional annotations of these DEGs were achieved by using Mercator tool (Mercator V3.6). Results of differential expression showed that out of 773 differentially expressed genes, 109 (13.05%) BINs assigned to protein process. The number of genes were assigned to the functional category “protein” was highest (103), followed by “RNA” (74), “stress” (52) and “misc” (42) (Fig. 5). Lastly, In the “O-64.1.10”, 82 out of 103 DEGs involved in protein processing (Fig. 5), were up-regulated compared to the wild-type (Table 4). Elongation factor 1-alpha (EF1α) play a crucial role in the protein synthesis machinery of cells, and its function extends to various physiological processes in plants, including wheat^48^. Our results indicated that this gene were highly expressed in “O-64.1.10” genotype.

**Table 4:** Categorization of differentially expressed hub genes (up/down-regulated) in “O-64.1.10” at 15 DPA into protein process; BIN code 29.

Code BIN	Class	No. BIN	No. Unigene	Up-regulated in O-64-1-10	down-regulated in O-64-1-10
29.1	Protein.aa activation	3	3	3	0
29.2	Protein.synthesis	26	23	18	5
29.3	Protein.targeting	9	9	7	2
29.4	Protein.postranslational modification	25	23	17	6
29.5	Protein.degradation	41	40	33	7
29.6	Protein.folding	4	4	3	1
29.7	Protein.glycosylation	1	1	1	0
Total	–	109	103	82	21

### Transcription factor, protein kinases and transcriptional regulators analysis

**Figure 6:**
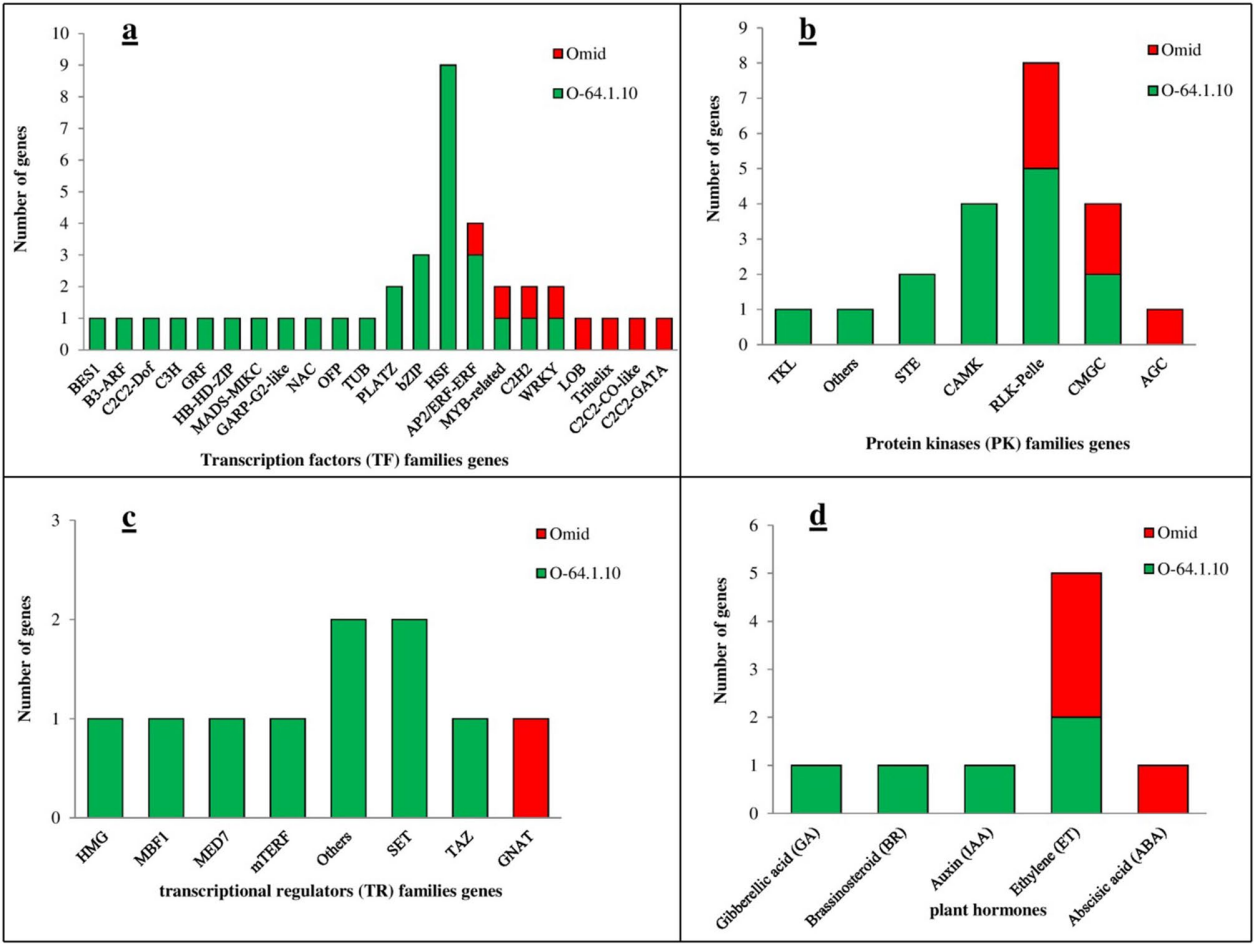
Distribution of transcription factor (TF) genes of 22 TF families (**a**), protein kinases (PK) genes of seven PK families (**b**), transcriptional regulators (TR) genes of eight families (**c**), and plant hormones (**d**) in comparison between Omid and O-64-1-10 genotypes with different baking quality at 15 DPA.

From DEGs, it was found that out of 22 TF families that the highest expression occurred for heat shock factor (HSF), AP2/ERF-ERF and basic leucine zipper (bZIP). Additionally, the HSF, bZIP, C2C2-Dof, B3-ARF, BES1, C3H, GRF, HB-HD-ZIP, PLATZ, MADS-MIKC, GARP-G2-like, NAC, OFP, and TUB TF families were exclusively expressed in the "O-64.1.10" genotype at the 15 DPA stage (Fig. 6a, supplemental data 3). Our findings revealed that, the bZIP TFs was related to storage protein gene regulation. TFs of the bZIP family are regulators of many central developmental and physiological processes including storage protein gene regulation^49^, energy metabolism^37^, light responses and oxidative stress signaling^50^. The regulations of the grain-filling process may be regulated by several TF families, such as bZIP and HSF TFs. The HSF TFs are regulators in response to stress^51^, early grain filling^16^, seed maturation^52^ and pollen development^53^. The HSF family is a transcriptional activator of heat shock protein (HSP) genes^54^. The HSP family is an important factor ensuring correct protein folding that plays a significant role in degradation pathways, such as endoplasmic reticulum-associated degradation^55^. Our results showed that LMW-GS genes and ERF TF had a higher expression in “Omid” and “O-64.1.10”, respectively. Hasrak et al.^56^ reported there is a negative correlation between ERF TFs and lower expression of LMW-GS. Our data indicated that the NAC TF (TraesCS2B01G359200.1) were found from highly expressed in “O-64.1.10” genotype. The NAC019 TF regulates glutenin and starch accumulation and its elite allele improves wheat grain quality^39^. The TKL, STE, CAMK, and Others of PKs were found from highly expressed in the “O-64.1.10” genotypes, whereas the AGC of PK families was expressed only in the “Omid” genotypes (Fig. 6b, supplemental data 3). The PKs may cooperate with the transcriptional networks to refine the regulation of genes during seed development^34^. In this study, among all genes with differential expression, seven important families of TRs such as HMG, MBF1, MED, mTERF, SET, TAZ, and others genes were found from highly expressed in the “O-64.1.10” genotype. In contrast, one family of TRs (GNAT) only was found from highly expressed in the wild-type genotype (Fig. 6c, supplemental data 3).

### Plant hormones and transposable elements (TEs) analysis

Among of the five hormone-related genes that were up-regulated in each of genotypes, three genes associated with gibberellic acid, brassinosteroid, and auxin were expressed only in “O-64.1.10” genotypes (Fig. 6d, supplemental data 3). The plant hormones regulate multiple biological processes in early grain development of wheat and the comprehensive expression profiling provides useful information for hormone regulatory mechanisms^16^. The hormone response genes affect the wheat grain quality through indirect pathways^57^.

Our research displayed eleven TEs that were expressed only in “O-64.1.10” genotypes. These genes related to transposon may be responsible for causing the high baking quality in the mutant genotype “O-64.1.10”. So, further research is needed to find mutations governing genes related baking quality. TEs are mobile genetic elements in the eukaryotic genome which alter the expression of neighboring genes via insertion into promoter regions, or disrupt the function of protein-coding genes when inserted into the genes, or even change gene structure by altering its splicing and polyadenylation patterns^58–60^.

### Identification of SSRs

In the context of differential gene expression between the “O-64.1.10” and wild-type genotypes, 127 SSRs were identified. The repeat pattern of the identified SSRs indicated a prevalence of trinucleotide repeats (122) were the most abundant, followed by di- (3) and hexa- (2) nucleotide repeats (Table 5, supplemental data 3). Within the trinucleotide repeats, CCG/CGG (54) was the most frequent motif, followed by AGG/CCT (26) and AGG/CCT (22). The majority of motifs (96.06%) consisted of 5–7 repeats, while motifs with 8–10 repeats were rare (3.94%). For instance, gamma-gliadin contained AAC motifs with 10 repeats. A total of 109 sequences containing SSRs, 17 sequences containing more than one SSR, and 8 sequences containing SSRs in compound formation were identified. For instance, alpha-gliadin contained (ACA)9 and (CAA)8 motifs. These genic SSRs/RNAseq-SSR markers were developed from transcriptome sequences and can be used for marker-assisted selection (MAS).

**Table 5:** Details of SSR markers obtained from DEGs of “Omid” and “O-64.1.10”.

	Omid	O-64.1.10	Total
Total number of sequences examined	189	584	773
Total number of identified SSRs	27	100	127
Number of SSR containing sequences	27	82	109
Number of sequences containing more than 1 SSR	0	17	17
Number of SSRs present in compound formation	0	8	8
Mono	0	0	0
Di	0	3	3
Tri	26	96	122
Tetra	0	0	0
Penta	0	0	0
Hexa	1	1	2

### Quantitative Reverse Transcription PCR Validation

**Figure 7:**
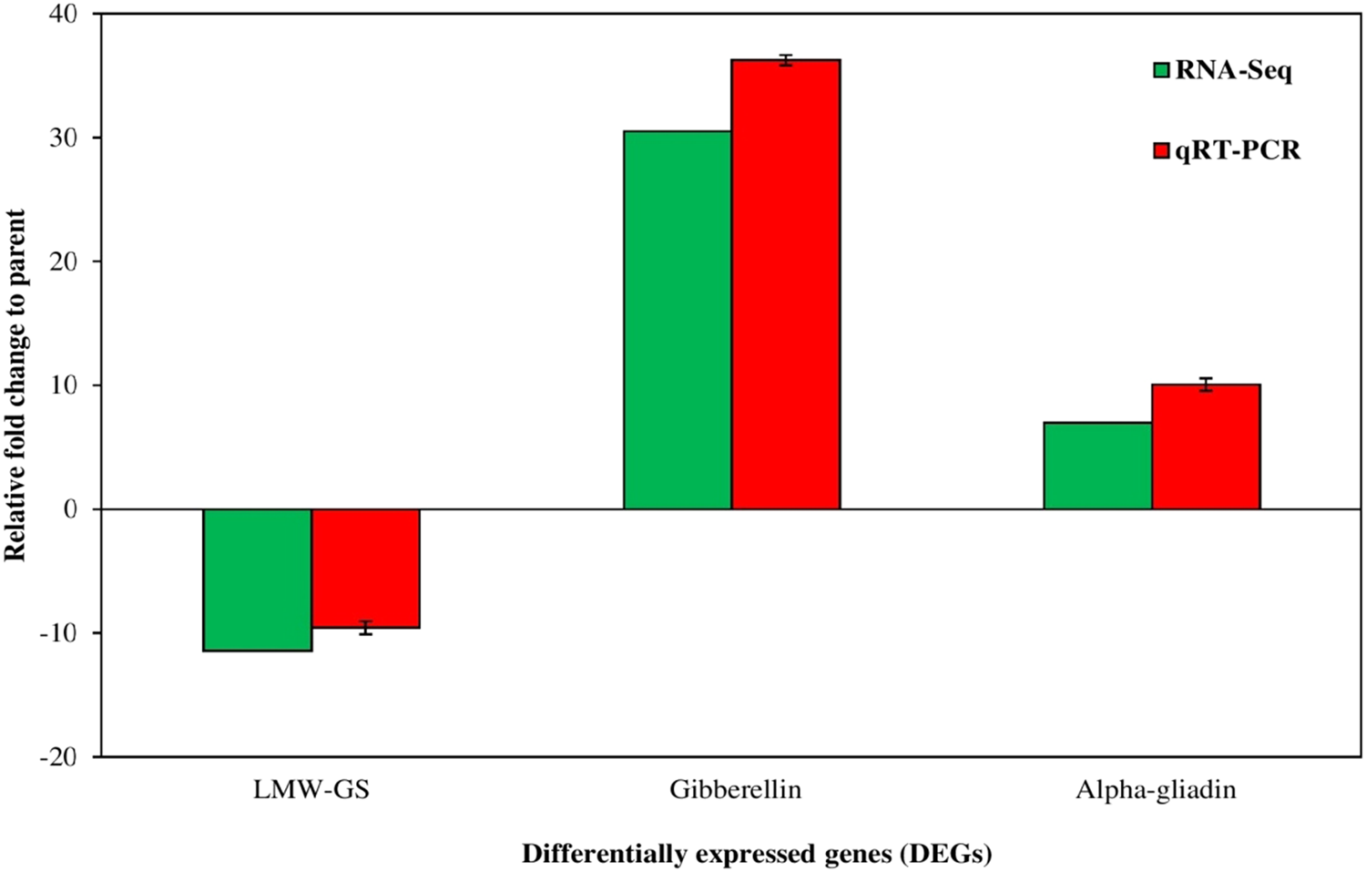
Quantitative Reverse Transcription PCR validation of RNA-seq results for selected DEGs.

qRT-PCR was used to validate the RNA-seq results. Therefore, out of DEGs, three important genes involved in baking quality were selected from the DEGs, including LMW-GS, Alpha-gliadin and Gibberellin. The LMW-GS and gliadins (alfa/beta, omega, and gamma) are the main storage proteins and the major components of the gluten polymer. The accumulation of these storage proteins affects wheat quality formation^17^. The relative fold changes in gene expression measured by qRT-PCR were found to be consistent with their expression levels determined by RNA-Seq. Both techniques yielded similar patterns of changes, validating the results obtained from RNA-Seq (Fig. 7).

## Conclusions

The transcriptomics analysis is a powerful tool to study molecular processes taking place during grain development. In this study, differential expressed genes of two breed wheat genotypes “O-64.1.10” and “Omid” were investigated at 15 DPA by transcriptomics approaches. a total number of 733 DEGs were identified between each genotype, as well as the DEGs analysis suggested that up-regulated genes in “O-64.1.10” were enriched on A-genome, whereas down-regulated genes in “O-64.1.10” were overexpression on B-genome. Our recent studies have identified several crucial factors that regulate the baking quality of wheat, including grain storage proteins (gluten), TFs, TRs, PKs, plant hormones, carbohydrates (starch and sucrose), lipids (unsaturated fatty acids), and vitamins. Using the KEGG pathway enrichment analysis of DEGs comparing “O-64.1.10” with “Omid” showed that various pathways of metabolic pathways, protein processing in endoplasmic reticulum, biosynthesis of secondary metabolites, spliceosome, ribosome, plant hormone signal transduction, and biosynthesis of amino acids were enriched in the “O-64.1.10” genotype with the highest number of the genes, whereas the pathways associated with metabolic pathways, biosynthesis of secondary metabolites, and ribosome were frequent in the “Omid” genotype. Yu et al.^17^ reported that metabolic pathway network analysis that major and minormetabolic pathways regulate one another to ensure regular seed development and nutritive reserve accumulation. Yan et al.^61^ showed that carbohydrate and hormone metabolism has play important roles in the grain size and weight in wheat.

Our results indicated that TraesCS2A01G483600 (encodes Elongation factor 1-alpha) expressed only in “O-64.1.10” genotypes. Paul et al.^48^ suggested that Elongation factor 1-alpha gene play an important role in cell expansion in early developmental wheat grain. The validation of transcripts via qRT-PCR presented in this study holds significant academic value. These transcripts have been found to have a positive correlation with the enhancement of the baking quality of wheat grains. Lastly, in addition, we also identified 127 RNA-seq SSR markers among all genes with differential expression in “O-64.1.10” and its wild type genotype. The repeat pattern of identified SSRs indicated an abundance of dinucleotides followed by tri, and hexanucleotide repeats.

In conclusion, results from our study provides valuable shed light into the genetic mechanisms underlying baking quality in wheat flour production, and could have significant implications for the baking industry.

### Supplementary Information

Supplementary Information.

## Data Availability

The datasets generated and/or analysed during the current study are available in the SRA NCBI repository, https://www.ncbi.nlm.nih.gov/sra/?term=PRJNA979921. BioSample accessions: https://www.ncbi.nlm.nih.gov/biosample/SAMN35615225. https://www.ncbi.nlm.nih.gov/biosample/?term=SAMN35615224.
